# Adaptation and Validation of the 14-Item Religious and Spiritual Struggles Scale Among Russian Orthodox Christian Women

**DOI:** 10.1007/s10943-026-02645-y

**Published:** 2026-04-09

**Authors:** Fedor Shankov, Maria Böttche, Annett Lotzin

**Affiliations:** 1 Department of Psychiatry and Psychotherapy, University Medical Center Hamburg-Eppendorf, Hamburg, Germany; 2 Division of Clinical Psychological Intervention, Freie Universität Berlin, Berlin, Germany; 3 Institute for Clinical Psychology and Psychotherapy, MSH Medical School Hamburg, Hamburg, Germany

**Keywords:** Religious/spiritual struggles, Validation, Adaptation, Measurement, Mental health, Russian Orthodox

## Abstract

Religious and spiritual (R/S) struggles are associated with psychological distress across cultures, yet no validated Russian-language instrument exists to assess these experiences in Orthodox Christian populations. This study adapted and validated the Religious and Spiritual Struggles Scale (RSS-14) for Russian Orthodox Christian women. Two samples of Russian Orthodox Christian women were recruited through Orthodox online platforms during Great Lent 2025: a cross-sectional sample (*N* = 593) and a longitudinal sample (*N* = 476) assessed over six weeks. Structural validity was evaluated using confirmatory factor analysis (CFA); reliability via internal consistency and test–retest stability; measurement invariance across timepoints; and construct validity through correlations with religiosity, distress, and well-being measures. CFA supported a six-factor structure (Divine, Demonic, Interpersonal, Moral, Doubt, Ultimate Meaning) with acceptable-to-good fit. Internal consistency was adequate and test–retest reliability moderate. Metric and scalar invariance were supported. Convergent validity was demonstrated through correlations with negative religious coping, and criterion validity through associations with depression, anxiety, stress, and reduced well-being. The findings indicate that the RSS-14 provides a valid and reliable tool for assessing R/S struggles among Russian Orthodox Christian women, addressing a critical gap in Russian-language instrumentation for research, clinical, and pastoral applications.

**Supplementary Information:**

The online version contains supplementary material available at 10.1007/s10943-026-02645-y.

## Introduction

While the health-promoting effects of religious and spiritual (R/S) engagement are well established (Koenig et al., 2024; Schwalm et al., 2022; Yaden et al., 2022), growing evidence documents that R/S can also be negatively associated with health and well-being—particularly through experiences of doubt, distress, or discord related to one’s faith, beliefs, or spiritual identity (Exline et al., 2024; Papaleontiou-Louca, 2024). These phenomena occur across every R/S tradition, affecting highly devout individuals as well as secular individuals and atheists (Pargament & Exline, 2021). The R/S struggles framework represents one of the most comprehensive and empirically grounded conceptualizations for understanding religious difficulties and related experiences.

### Definition and Measures of Religious and Spiritual Struggles

R/S struggles refer to experiences of tension, conflict, and strain centered on whatever a person views as sacred (Exline et al., 2023). They can be organized into three broad domains (supernatural, interpersonal, and intrapsychic) comprising six empirically derived subdomains. *Divine struggles* involve negative emotions or conflicts in one’s relationship with God, such as feeling punished or abandoned. *Demonic struggles* center on fears of evil forces or perceived spiritual attack. *Interpersonal struggles* refer to relational strains with religious community members, including feelings of hurt and conflict over faith matters. *Moral struggles* occur when individuals wrestle with guilt or ethical dilemmas related to falling short of their moral ideals. *Doubt-related struggles* involve profound questions and uncertainties about doctrine, belief, or God’s existence. *Ultimate meaning struggles* pertain to existential crises—feelings that life lacks deeper meaning or significance.

Research consistently links R/S struggles to poorer well-being and mental health problems, including depression, anxiety, suicidal ideation, and obsessive–compulsive symptoms (Bockrath et al., 2022; Exline et al., 2024; Smith et al., 2025). Emerging longitudinal evidence has provided the strongest support for the primary model, in which spiritual struggles predict subsequent psychological distress, though modest support has also emerged for the secondary model (distress leading to struggles) and bidirectional relationships; however, the primary model has been tested most often and more research is needed on the alternative models (Pargament et al., 2025).

Exline et al. (2014) developed the Religious and Spiritual Struggles Scale (RSS) to assess these six domains, with a briefer 14-item version (RSS-14) subsequently created (Exline et al., 2023). Both versions demonstrated good psychometric properties (Smith et al., 2025), and cross-cultural validations in Poland (Falewicz et al., 2023) and Portugal (Tomás & Moreira, 2024) replicated the factor structure and expected associations with mental health. More recently, Salcone et al. (2025) derived a five-item screening version (RSS-5) from the full 26-item RSS for rapid clinical use; however, this ultra-brief version employs a unidimensional structure that does not permit differentiation among struggle types.

### The Russian Orthodox Christian Context

Despite extensive validation of RSS measures in Western contexts, significant gaps remain in understanding how R/S functions in non-Western cultural and linguistic contexts (Herzog, 2020; Richards et al., 2021). Russian ranks as the seventh most widely spoken language globally and the most widely spoken in Eurasia (Eberhard et al., 2024), with Orthodox Christianity representing the dominant religious tradition, comprising over 100 million adherents in Russia alone. Together with populations in Ukraine, Belarus, and other former Soviet Union countries, the Russophone Orthodox community constitutes nearly half of the world’s Eastern Orthodox Christians (Pew Research Center, 2017). To date, no validated Russian-language instrument captures the multidimensional nature of R/S struggles, nor have any methodologically rigorous empirical studies examined these experiences within Russian-speaking Orthodox Christian populations—a significant gap given recent geopolitical and societal upheavals affecting this community (Fornaro et al., 2025; Kolstø, 2025).

In a nationally representative Russian sample (*N* = 1600), approximately two-thirds of adults identify as Russian Orthodox, yet only 14% attend services regularly and merely 23% associate religion with personal communion with God—most view it as national tradition or moral norms; consequently, only 7% report that religion regularly helps in everyday life (Russian Public Opinion Research Center, 2025). Cross-national data corroborate this pattern: religiosity showed no association with self-reported health in Russia and other post-communist societies, unlike the United States and South Africa (Zimmer et al., 2019). This identification-practice gap has been attributed to “ethnodoxy”—ethnic identity linked to tradition without corresponding spiritual depth (Karpov et al., 2012)—though insufficient clergy for individual pastoral care may also constrain engagement despite spiritual demand (Emelyanov, 2018). Contemporary Russian Orthodox religiosity thus often blends pre-revolutionary traditions with folk beliefs and Soviet-era elements (Pew Research Center, 2018).

Eastern Orthodoxy differs from Western Christian traditions in its emphasis on liturgical participation, sacramental mystery, ascetical discipline, and communal ecclesial authority (Mitralexis & Kaethler, 2023). The Orthodox focus on continual repentance (*metanoia*) and spiritual warfare provides a culturally sanctioned framework for moral and demonic struggles that differs from Western framings. Gender dynamics warrant attention: lay women bear central roles in sustaining Russian Orthodox practice and its emotional labor (Spivak & Seyidova, 2024; Tocheva, 2021) while facing structural exclusion (Beliakova, 2025)—a combination suggesting heightened vulnerability to religious struggles with limited resources to address them.

Despite practitioner descriptions of phenomena resembling R/S struggles (Filonik, 2016; Gedevani et al., 2022; Velikanov, 2022), no methodologically rigorous studies using validated instruments have examined these experiences in Russian-speaking Orthodox populations.

### The Present Study

Given the absence of validated Russian-language R/S struggles measures, the cultural and theological distinctiveness of Russian Orthodoxy, and contemporary challenges facing this population, a psychometrically sound tool is needed. R/S constructs are culturally sensitive, necessitating meticulous translation, expert evaluation, and empirical testing (Büssing, 2025; Koenig & Al Zaben, 2021; Richards et al., 2021). This study aimed to adapt and validate the RSS-14 for Russian Orthodox Christian women following recommended psychometric guidelines (Cruchinho et al., 2024; Gagnier et al., 2021; Koenig & Al Zaben, 2021).

## Method

### Participants

This study comprised two independent samples of Russian Orthodox Christian women recruited through Orthodox church communities and online platforms in Russia during March–April 2025, coinciding with Great Lent—a period of heightened religious engagement (intensified fasting, penitential prayer, increased participation in Lenten services, and repentance) particularly suited to assessing R/S experiences. Inclusion criteria were: (a) informed consent, (b) self-identification as female, (c) age ≥ 18 years, (d) affiliation with the Russian Orthodox Church, and (e) reading and writing in Russian. Detailed sociodemographic characteristics are presented in Table 1.

**Table 1:** Participant sociodemographic characteristics

Characteristic	Sample 1 (*N* = 593)	Sample 2 (*N* = 476)		
		T1 (*N* = 355)	T2 (*N* = 307)	T3 (*N* = 251)
Age, years *M* (*SD*)	46.89 (9.67)	43.81 (11.99)	44.16 (12.01)	44.27 (12.14)
*Marital status n* (%)				
Married/cohabiting	349 (58.9%)	222 (62.5%)	191 (62.2%)	155 (61.8%)
Single/divorced/widowed	234 (39.5%)	125 (35.2%)	112 (36.5%)	92 (36.7%)
Missing/No response	10 (1.7%)	8 (2.3%)	4 (1.3%)	4 (1.6%)
*Parental status n* (%)				
Has children (≥ 1)	443 (74.7%)	241 (67.9%)	215 (70.0%)	171 (68.1%)
No children	143 (24.1%)	110 (31.0%)	88 (28.7%)	76 (30.3%)
Missing/No response	7 (1.2%)	4 (1.1%)	4 (1.3%)	4 (1.6%)
*Education level n* (%)				
Basic	4 (0.7%)	1 (0.3%)	0 (0.0%)	0 (0.0%)
General secondary	12 (2.0%)	9 (2.5%)	8 (2.6%)	7 (2.8%)
Vocational secondary	70 (11.8%)	25 (7.0%)	24 (7.8%)	14 (5.6%)
Incomplete higher	25 (4.2%)	18 (5.1%)	17 (5.5%)	12 (4.8%)
Higher	370 (62.4%)	227 (63.9%)	188 (61.2%)	163 (64.9%)
Multiple higher/postgraduate	86 (14.5%)	74 (20.8%)	69 (22.5%)	54 (21.5%)
Missing/No response	26 (4.4%)	1 (0.3%)	1 (0.3%)	1 (0.4%)
*Employment status n* (%)				
Full-time	211 (35.6%)	4 (1.1%)	5 (1.6%)	4 (1.6%)
Part-time	240 (40.5%)	26 (7.3%)	21 (6.8%)	18 (7.2%)
Unemployed, seeking	39 (6.6%)	20 (5.6%)	15 (4.9%)	13 (5.2%)
Not in labor force	55 (9.3%)	15 (4.2%)	11 (3.6%)	10 (4.0%)
Retired	32 (5.4%)	112 (31.5%)	87 (28.3%)	71 (28.3%)
Disabled/not working	8 (1.3%)	146 (41.1%)	137 (44.6%)	114 (45.4%)
Missing/No response	8 (1.3%)	32 (9.0%)	31 (10.1%)	21 (8.4%)
*Socioeconomic status n* (%)				
Extreme poverty	14 (2.4%)	1 (0.3%)	1 (0.3%)	1 (0.4%)
Low income	106 (17.9%)	31 (8.7%)	27 (8.8%)	19 (7.6%)
Lower-middle	148 (25.0%)	147 (41.4%)	116 (37.8%)	106 (42.2%)
Upper-middle	257 (43.3%)	134 (37.7%)	128 (41.7%)	95 (37.8%)
High income	31 (5.2%)	12 (3.4%)	13 (4.2%)	11 (4.4%)
Missing/No response	37 (6.2%)	30 (8.5%)	22 (7.2%)	19 (7.6%)
*Country/region of residence n* (%)				
Russia	491 (82.8%)	306 (86.2%)	263 (85.6%)	216 (85.9%)
Other post-Soviet states	56 (9.4%)	33 (9.3%)	26 (8.5%)	20 (8.0%)
Other countries	46 (7.8%)	16 (4.5%)	18 (5.9%)	15 (6.0%)

*M* = mean; *SD* = standard deviation. Cells show *N* = number of participants and % = percentages calculated from column *N*, including Missing/No response. Sample 2 columns correspond to measurement waves T1–T3

#### Sample 1 (Cross-Sectional)

The cross-sectional sample comprised 593 women aged 18–70 years (*M* = 46.89, *SD* = 9.67). Most were married or cohabiting (58.9%), had at least one child (74.0%), held higher education degrees (76.9%), and were currently employed (76.1%). Participants predominantly resided in Russia (82.8%), with smaller proportions from Ukraine, Belarus, and other countries.

#### Sample 2 (Longitudinal)

The longitudinal sample comprised 476 participants, with participation rates of *N* = 355 (74.6%) at T1, *N* = 307 (64.5%) at T2, and *N* = 251 (52.7%) at T3. At T1, participants were aged 18–78 years (*M* = 43.81, *SD* = 11.99). Most were married or cohabiting (62.5%), had at least one child (67.9%), held higher education degrees (84.7%), and were employed (72.6%). Geographic distribution was similar to Sample 1, with the majority residing in Russia (86.2%).

### Procedure

Both samples were recruited through voluntary, self-selected participation on online Orthodox platforms, constituting non-probability convenience samples. Sample 1 participants were recruited through “Psychology with Marina Filonik” (*Psikhologiya s Marinoy Filonik*) and the related “Psychology for the Church” (*Psikhologiya dlya tserkvi*)—Russian-language platforms providing psychological education integrated with Orthodox Christian perspectives. Sample 2 participants were recruited through “Life with Meaning” (*Zhizn’ so smyslom*), an online educational platform offering Christian-oriented courses and programs.

Sample 1 (cross-sectional) participants were provided with a full description of the study purposes and informed consent before participation. Participants completed an online survey hosted on the GDPR-compliant 1KA platform. The survey was pilot-tested for technical functionality prior to data collection and was publicly accessible. Participants could skip or decline any question, though gentle reminders encouraged completion to facilitate accurate scoring and feedback generation. Upon completion, participants received a personalized feedback report summarizing their individual scores on the main study measures, general population norms, brief explanations of assessed constructs, and general evidence-based self-help recommendations for enhancing mental health and mindfulness. In addition, participants were offered psychoeducational materials and an optional promotional code for a free trauma-informed online seminar.

Sample 2 (longitudinal) participants were recruited through an online psychoeducational program for Russian Orthodox Christians observing Great Lent. The program utilized the GetCourse learning platform, where each participant maintained a personal account housing all study materials and survey assessments. Data were collected using the platform’s integrated survey tool, which enabled export of responses with unique participant identifiers to ensure accurate longitudinal matching across measurement waves.

At each wave, participants provided informed consent before completing assessments embedded within the program curriculum. To encourage retention, participants received modest incentives (approximately USD 2 in platform credits) and, upon completing all three waves, personalized feedback reports.

The study was approved by the Ethics Committee for Psychological and Pedagogical Research, Moscow City Pedagogical University (Nos. 021-2023, 038-2025).

### Translation and Cultural Adaptation of the RSS-14

After obtaining permission from the original authors (Exline et al., 2023), a Russian version was developed following established guidelines (Beaton et al., 2000). Forward translation was conducted by two certified translators and one professional psychologist independent of the study team. Two bilingual mental health professionals performed back-translation, and discrepancies were resolved through consensus.

The reconciled version underwent two rounds of expert evaluation. The first panel (*N* = 16) included Orthodox Christian psychologists, priests, scholars, and monastics; the second panel (*N* = 19) included psychologists, priests, lay church members, and religious studies researchers. Experts rated item relevance and clarity, and the scale-level Content Validity Index (S-CVI) reached acceptable levels (≥ 0.82). Items receiving low item-level CVI ratings were iteratively refined based on expert recommendations. The adapted version was pilot-tested (*N* = 46), and psychometrically weak items were further improved. The final back-translation received approval from the original scale author. For original and translated item wordings, see Table 2.

**Table 2:** RSS-14 item wording: original English, Russian adaptation, and back-translation

Item	Original English	Russian adaptation	Back-translation
Do1	Felt troubled by doubts or questions about religion or spirituality	Пepeживaл(a) cмятeниe и тpeвoгy из-зa coмнeний в вepe или дyxoвнoй жизни	Experienced confusion and anxiety due to doubts in faith or spiritual life
Di1	Felt angry at God	Иcпытывaл(a) чyвcтвo oбиды или нeгoдoвaния пo oтнoшeнию к Бoгy	Felt hurt feelings or indignation toward God
IP1	Felt hurt, mistreated, or offended by religious/spiritual people	Пepeживaл(a), чтo cлyжитeли Цepкви или дpyгиe вepyющиe плoxo co мнoй oбoшлиcь, ocкopбили или oбидeли мeня	Experienced, that religious people or Church ministers hurt me, treated me poorly, or insulted me
De1	Felt attacked by the devil or by evil spirits	Oщyщaл(a), чтo бecы или дьявoл иcкyшaют или нaпaдaют нa мeня	Sensed that demons or the devil were tempting or attacking me
Me1	Questioned whether life really matters	Зaдaвaлcя(-acь) вoпpocoм, дeйcтвитeльнo ли мoя жизнь имeeт cмыcл	Questioned whether my life really has meaning
IP2	Had conflicts with other people about religious/spiritual matters	Кoнфликтoвaл(a) или cпopил(a) c дpyгими людьми пo вoпpocaм вepы и дyxoвнoй жизни	Conflicted or argued with other people on matters of faith and spiritual life
Mo1	Wrestled with attempts to follow my moral principles	Бopoлcя(-acь) c coбoй, в пoпыткax cлeдoвaть мoим нpaвcтвeнным пpинципaм	Struggled with myself in attempts to follow my moral principles
IP3	Felt angry at organized religion	Пepeживaл(a) нecoглacиe или paздpaжeниe пo пoвoдy ycтpoйcтвa жизни Цepкви или пocтyпкoв eё cлyжитeлeй	Experienced disagreement or irritation regarding the organization of Church life or the actions of its ministers
Do2	Felt confused about my religious/spiritual beliefs	Иcпытывaл(a) нeyвepeннocть или кoлeбaлcя(-acь) в cвoиx peлигиoзныx yбeждeнияx	Experienced uncertainty or wavered in my religious beliefs
Di2	Felt as though God was punishing me	Пepeживaл(a), cлoвнo Бoг мeня нaкaзывaeт	Experienced as if God was punishing me
De2	Worried that the problems I was facing were the work of the devil or evil spirits	Oщyщaлa(a), чтo тpyднocти, c кoтopыми я cтaлкивaюcь,—этo пpoиcки бecoв или дьявoлa	Experienced that the difficulties I face are the deeds of demons or the devil
Di3	Felt as though God had abandoned me	Пepeживaл(a) oщyщeниe, cлoвнo Гocпoдь ocтaвил мeня	Experienced a sensation as if the Lord had abandoned me
Me2	Felt as though my life had no deeper meaning	Пepeживaл(a), чтo мoя жизнь лишeнa ocoбoгo cмыcлa	Experienced that my life lacks special meaning
Mo2	Felt guilty for not living up to my moral standards	Иcпытывaл(a) винy зa тo, чтo нe yдaeтcя жить coглacнo cвoим нpaвcтвeнным пpинципaм	Felt guilt for not being able to live according to my moral principles

Items are presented in the order of the scale and their subscale assignments: De = Demonic Struggles; Di = Divine Struggles; Do = Doubt Struggles; IP = Interpersonal Struggles; Me = Ultimate Meaning Struggles; Mo = Moral Struggles. Item wording was culturally adapted for Russian Orthodox Christian context

### Measures

Participants completed demographic and self-report measures. Descriptive statistics and internal consistency are presented in Table S1.

#### Religiosity/Spirituality Measures

*Religious and Spiritual Struggles Scale-14 (RSS-14)*. The RSS-14 (Exline et al., 2023) assesses six domains of R/S struggles: divine, demonic, interpersonal, moral, doubt, and ultimate meaning. Items are rated on a 5-point Likert-type scale ranging from 1 (*not at all*) to 5 (*a great deal*), reflecting experiences over a specified timeframe (one month for Sample 1; two to three weeks for Sample 2). Subscale scores are computed as item means. The original English RSS-14 demonstrated a robust six-factor structure and good internal consistency across subscales (α = 0.75–0.88; Exline et al., 2023). Psychometric properties of the Russian Orthodox adaptation are the focus of the present study.

*Brief Religious Coping Scale (Brief RCOPE)*. The Brief RCOPE (Pargament et al., 2011) assesses Positive Religious Coping (e.g., “Looked for a stronger connection with God”) and Negative Religious Coping (e.g., “Wondered whether God had abandoned me”) in response to recent stressors, rated from 0 (*not at all*) to 3 (*a great deal*). The Russian version (Shankov et al., 2022) comprises 7 Positive and 6 Negative Religious Coping items, demonstrates good internal consistency, and predicts adjustment in religious samples. The present study employed a Russian translation retaining the original response format (4-point extent scale). Sample 1 received simplified situational instructions (coping with a specific recent stressor), whereas Sample 2 received dispositional instructions (coping with major life problems in general). In Sample 1, Positive Coping α = 0.84 (ω = 0.92) and Negative Coping α = 0.81 (ω = 0.93); in Sample 2 (T1), Positive Coping α = 0.83 (ω = 0.91) and Negative Coping α = 0.75 (ω = 0.90).

*Centrality of Religiosity Scale-5 (CRS-5)*. The CRS-5 (Huber & Huber, 2012) measures the salience of religion across five dimensions (e.g., “How often do you pray?”) using dimension-specific rating scales. The Russian CRS-5 demonstrates good validity and modest internal consistency, as items assess distinct facets of religiosity (Ackert et al., 2020). In Sample 1, α = 0.64 (ω = 0.84); in Sample 2 (T1), α = 0.61 (ω = 0.81).

*Daily Spiritual Experiences Scale—Short Form (S-DSES)*. The S-DSES (Underwood & Teresi, 2002) assesses frequency of daily spiritual experiences (e.g., “I feel God’s presence”), rated from 1 (*never*) to 6 (*many times a day*). Sample 1 completed the 7-item Russian version, whereas Sample 2 completed only a reduced 4-item theistic version (reflecting divine closeness experiences), which excludes items potentially conflated with mental health outcomes (Shankov et al., 2025). Scores are averaged; higher scores indicate more frequent spiritual experiences. In Sample 1, α = 0.88 (ω = 0.93); in Sample 2 (T1), α = 0.83 (ω = 0.88).

#### Psychological Distress

*Depression Anxiety Stress Scales-21 (DASS-21)*. The DASS-21 (Lovibond & Lovibond, 1995) measures psychological distress via three 7-item subscales: Depression, Anxiety, and Stress, rated from 0 (*did not apply*) to 3 (*applied very much or most of the time*) over the past week. The validated Russian version (Zolotareva, 2021) demonstrates high internal consistency and validity across diverse samples. In Sample 1, the General Distress total score showed excellent reliability (α = 0.93, ω = 0.96), as did the subscales: Depression (α = 0.86, ω = 0.92), Anxiety (α = 0.85, ω = 0.92), and Stress (α = 0.89, ω = 0.95).

*Patient Health Questionnaire-4 (PHQ-4)*. The PHQ-4 (Kroenke et al., 2009) assesses depression (2 items) and anxiety (2 items) over the past 2 weeks, rated from 0 (*not at all*) to 3 (*nearly every day*). The Russian version (Zolotareva et al., 2024) was administered at all three waves in Sample 2. Spearman–Brown reliability coefficients were 0.83 for Depression and 0.89 for Anxiety.

*Perceived Stress Scale-4 (PSS-4)*. The PSS-4 (Cohen et al., 1983) assesses perceived stress over the past month on a 5-point scale from 0 (*never*) to 4 (*very often*). The Russian PSS-4 (Zolotareva, 2023) demonstrates acceptable reliability and convergent validity with health outcomes. In Sample 2 (T1), α = 0.78 and ω = 0.87.

#### Well-Being

*Satisfaction With Life Scale (SWLS)*. The SWLS (Diener et al., 1985) measures global life satisfaction via 5 items rated from 1 (*strongly disagree*) to 7 (*strongly agree*). The Russian SWLS (Osin & Leontiev, 2020) demonstrates strong reliability and a unidimensional structure. In the current study, Sample 1, α = 0.81 and ω = 0.86.

*WHO-5 Well-Being Index*. The WHO-5 (World Health Organization, 1998) assesses subjective well-being over the past 2 weeks via 5 items rated from 0 (*at no time*) to 5 (*all of the time*). Raw scores (0–25) are multiplied by 4 to yield a 0–100 metric. The Russian version (Topp et al., 2015) was administered at all waves in Sample 2 as the primary well-being outcome. The WHO-5 demonstrates good validity as a screening tool. In Sample 2 (T1), α = 0.88 and ω = 0.93.

### Data Preparation and Assumptions

Data were screened for quality and completeness. The number of male participants in both samples was insufficient for meaningful statistical analysis, making their inclusion unrepresentative. The same exclusion criteria were applied across both datasets: duplicate records, ineligible respondents (age < 18, male, or not Russian Orthodox), careless responding (≥ 2 indicators: high modal responding, long identical-response strings, or minimal variability), speeders completing the survey in less than one-third of the median duration, and cases with extensive missing data (> 20% on key measures). Multivariate outliers identified using Mahalanobis distance (*p* < 0.001) were removed.

For Sample 1, the online survey received 1923 visitors, of whom 637 completed the RSS-14 and validity measures; after applying exclusion criteria and data cleaning, a total of 593 participants were retained. For Sample 2, the pre-study survey yielded 1099 visitors, of whom 521 participated in at least one subsequent wave; after data cleaning, a total of 476 participants were retained.

Item-level missingness was minimal across both samples, not exceeding 5.6% for any variable. For Sample 2, longitudinal data exhibited expected attrition: of all possible values, 31.2% were missing across waves, increasing from 0.4% at pre-study to 25.2% (T1), 34.9% (T2), and 47.3% (T3); item-level refusal rates remained below 0.8% at each wave. Given low missingness rates and the use of pairwise present estimation under WLSMV (Weighted Least Squares Means and Variance Adjusted) in lavaan (Rosseel, 2012), missingness was treated as ignorable, retaining all available information without listwise deletion (Table S2).

Univariate and multivariate distributions were examined prior to analysis. All RSS-14 items showed positive skew, consistent with community samples where most participants report few or no struggles (Exline et al., 2023). Because this reflected meaningful floor effects rather than measurement error, no transformations were applied. Confirmatory factor analyses used WLSMV estimation to accommodate ordinal indicators and non-normality.

### Analytic Plan

#### Confirmatory Factor Analysis

To test the factor structure of the RSS-14, we conducted Confirmatory Factor Analysis. Given the ordinal Likert responses and non-normal distribution of items, we used a robust WLSMV estimator with theta parameterization appropriate for categorical data (Li, 2016). The hypothesized model specified six correlated latent factors (Divine, Demonic, Interpersonal, Moral, Ultimate Meaning, Doubt), each loading on its respective 2–3 items (Exline et al., 2023). Model fit was evaluated using conventional indices: chi-square (χ^2^), degrees of freedom (*df*), chi-square to degrees of freedom ratio (χ^2^/*df*), Comparative Fit Index (CFI), Tucker–Lewis Index (TLI), Root Mean Square Error of Approximation (RMSEA), and Standardized Root Mean Square Residual (SRMR).

We followed conventional cutoff guidelines (e.g., CFI/TLI ≥ 0.90 for acceptable fit, ≥ 0.95 for good fit; RMSEA ≤ 0.08 acceptable, ≤ 0.06 good; SRMR ≤ 0.08; Hu & Bentler, 1999). However, following recent recommendations by McNeish and Wolf (2023), we also computed dynamic fit indices (DFI) using Monte Carlo simulation (500 replications). DFI cutoffs were derived by simulating data from the fitted model with incrementally added cross-loadings to represent minor misspecifications. We extracted the 5th percentile of SRMR and RMSEA and the 95th percentile of CFI from the simulated distributions. This approach accounts for model complexity and sample size, avoiding overly rigid application of fixed thresholds.

#### Reliability Analyses

We evaluated internal consistency for each subscale. In addition to conservative Cronbach’s α, McDonald’s ω was calculated via polychoric correlation matrices when appropriate (for ordinal data), providing a more robust estimate of composite reliability that accounts for varying item-factor relationships. For two-item subscales (Demonic, Moral, Doubt), we report the more appropriate Spearman-Brown coefficient (Eisinga et al., 2013). Coefficients ≥ 0.70 were interpreted as acceptable.

Test–retest reliability of the RSS-14 was assessed via intraclass correlation coefficients (ICCs) and nonparametric Spearman’s rank correlation (*ρ*) between timepoints. Given the ordinal nature of the RSS-14, we report two-way mixed-effects, absolute-agreement, single-measure ICC(A,1) for 2-week (T1–T2) and 6-week (T1–T3) intervals. We also computed Bland–Altman mean differences and 95% limits of agreement to quantify absolute change and identify proportional bias. Standard error of measurement (SEM) was derived from the residual mean square of ICC models, and the smallest detectable change at 95% confidence (SDC95) was calculated as SEM × 1.96 × √2. Supplementary random-intercept linear mixed models spanning three occasions yielded ICC(A,1) estimates to assess consistency across all waves simultaneously.

#### Measurement Invariance

We examined whether the RSS-14 measurement model was invariant over time in Sample 2 (T1 vs. T2 vs. T3). Multi-group CFA was conducted in lavaan using the WLSMV estimator with robust corrections. Prior to analysis, we examined univariate category frequencies for each indicator at each wave. Adjacent response categories with frequencies below 5 observations per wave across two or more waves were systematically collapsed to ensure adequate cell frequencies for stable threshold estimation.

We tested a sequence of increasingly constrained models: (1) Configural invariance: same six-factor structure with freely estimated loadings across groups/timepoints; (2) Metric invariance: equal factor loadings across groups/timepoints; (3) Scalar invariance: equal loadings and thresholds (intercepts for ordinal data) across groups/timepoints; and (4) Strict invariance: equal loadings, thresholds, and residual variances across groups/timepoints.

Following Liu et al. (2017) recommendations for ordered-categorical CFA models, we used scaled chi-square difference tests to evaluate invariance at each step. A nonsignificant Δχ^2^ (*p* ≥ 0.05) was taken as evidence supporting invariance. Recognizing that chi-square tests can be overly conservative with ordered categorical data and dependent on sample sizes (Sass et al., 2014), we also examined changes in practical fit indices (ΔCFI, ΔRMSEA, ΔSRMR) as supplementary evidence. Following Chen (2007), we adopted the criteria of ΔCFI < 0.010 and ΔRMSEA < 0.015 to establish practical invariance when statistical tests were ambiguous. When invariance criteria were not met at any level, we examined modification indices to identify problematic items and explored partial invariance by freeing constraints on specific parameters, limiting such modifications to a maximum of 3 parameters to avoid overfitting.

#### Construct Validity

We assessed the convergent and divergent validity of the RSS-14 via nonparametric Spearman’s rank correlations with external criterion measures. In Samples 1 and 2, the key criteria were Negative Religious coping (Brief RCOPE subscale). We also analyzed psychological distress (DASS-21, PHQ-4, PSS-4), well-being (SWLS, WHO-5), and centrality of religiosity (CRS-5). Correlations were computed using pairwise complete observations. Significance was evaluated using two-tailed tests. We interpreted effect sizes per conventional benchmarks for Pearson correlations (*r* ≈ 0.10 small, 0.30 medium, 0.50 large), recognizing that these guidelines are also applicable to Spearman’s *ρ* (Akoglu, 2018).

All statistical analyses were conducted in R (version 4.5.1; R Core Team, 2025) using the lavaan package (Rosseel, 2012) for CFA and related packages for reliability and data management. Statistical analyses were independently verified by a qualified statistician external to the research team. During manuscript preparation, the authors used OpenAI GPT-5-Codex to refine R code and Anthropic’s Claude Sonnet 4.5 for language editing. All AI-assisted output was reviewed and verified by the authors, who take full responsibility for the final manuscript.

## Results

### Item Distributions

Item-level distributions demonstrated substantial non-normality across both samples, with most items exhibiting positive skewness (skew > 1) and excess kurtosis reaching approximately 9. This pattern of non-normality, characterized by floor effects and right-skewed distributions typical of spiritual struggles measures in predominantly religious samples, justified the use of WLSMV for all confirmatory factor analyses and measurement invariance tests. WLSMV is the recommended estimator for ordinal categorical data with non-normal distributions, as it provides unbiased parameter estimates and appropriate fit indices when standard maximum-likelihood estimation would be inappropriate (Liu et al., 2017). Detailed item-level descriptive statistics are presented in Table S3.

### Structural Validity

The RSS was originally conceptualized as a multidimensional scale assessing six distinct forms of R/S struggles (Exline et al., 2014, 2023). Consistent with this conceptualization, and given that a general factor was not supported in our data, we relied exclusively on the six subscale scores. Table 3 summarizes the global fit indices for the six-factor model across all samples.

**Table 3:** Confirmatory factor analysis model fit indices

	χ^2^ (*df*)	χ^2^*/df*	CFI	TLI	RMSEA [90% CI]	SRMR
Sample 1 (*N* = 593)	271.22 (62)	4.37	0.96	0.94	0.08 [0.07, 0.09]	0.05
Sample 2 (T1, *N* = 327)	148.13 (62)	2.39	0.96	0.94	0.07 [0.05, 0.08]	0.06
Sample 2 (T2, *N* = 293)	157.32 (62)	2.54	0.97	0.95	0.07 [0.06, 0.09]	0.07
Sample 2 (T3, *N* = 238)	97.02 (62)	1.56	0.99	0.98	0.05 [0.03, 0.07]	0.06
*Acceptable*	–	≤ 3.00	≥ 0.90	≥ 0.90	≤ 0.08	≤ 0.08
*Good*	–	≤ 2.00	≥ 0.95	≥ 0.95	≤ 0.06	≤ 0.06

Observed rows report WLSMV fit estimates for the RSS-14 with ordered categorical indicators and pairwise missing data handling. Threshold rows summarize conventional CFA cutoffs (Hu & Bentler, 1999). All χ^2^ tests have *p* < 0.01; χ^2^/*df* are reported for these tests. CFI = comparative fit index; TLI = Tucker-Lewis index; RMSEA = root mean square error of approximation; SRMR = standardized root mean square residual; χ^2^ = chi-square statistic

In Sample 1 (*N* = 593), the model showed acceptable-to-good fit. In the longitudinal Sample 2, model fit was adequate at the first wave and good across subsequent timepoints. Across all samples and timepoints, CFI and SRMR values consistently met the Hu and Bentler (1999) “good fit” benchmarks (CFI ≥ 0.95, SRMR ≤ 0.08), while TLI and RMSEA fell within the acceptable-to-good range (TLI ≥ 0.90, RMSEA ≤ 0.08). When evaluated against dynamic fit index (DFI) cutoffs tailored to the model characteristics (Table S4), all samples demonstrated fit consistent with correct model specification at Level 1 misspecification criteria.

### Factor Loadings

All loadings were statistically significant (*p* < 0.001). In Sample 1, loadings ranged from 0.53 (Interpersonal 2) to 0.95 (Ultimate Meaning 2), with item-level *R*^2^ values ranging from 0.28 to 0.89, indicating that items explained 28–89% of variance in their respective factors. All standardized loadings exceeded the 0.40 criterion recommended for adequate item–factor associations.

**Fig. 1:**
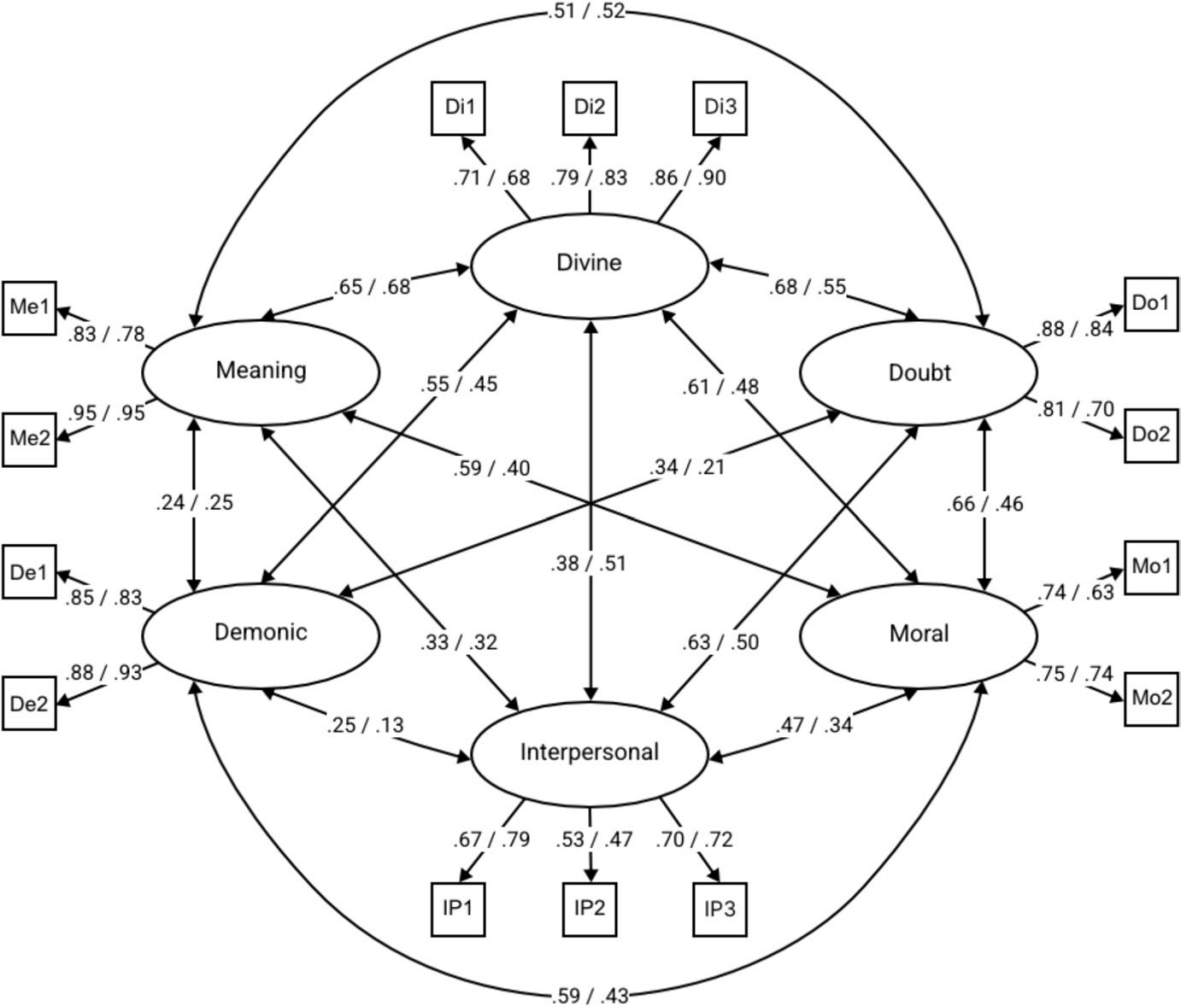
Path diagram of confirmatory factor analyses model: factor loadings and correlations. *Note*. Standardized CFA solution for RSS-14 using WLSMV estimation. Circles = latent factors; rectangles = observed items. Values show factor loadings (*λ*, single-headed arrows) and factor correlations (*Φ*, double-headed arrows) formatted as Sample 1 / Sample 2 (T1). All parameters *p* < 0.001

In Sample 2 (T1), loadings ranged from 0.47 (Interpersonal 2) to 0.95 (Ultimate Meaning 2); *R*^2^ values ranged from 0.22 to 0.90. One item (Interpersonal 2, λ = 0.47, *R*^2^ = 0.22) showed the lowest loading yet remained within acceptable limits for an initial cultural adaptation. Across both samples, the Ultimate Meaning subscale items yielded the strongest relations to their latent factor. Standardized factor loadings for all RSS-14 items are displayed in Fig. 1.

### Factor Correlations

Latent interfactor correlations are summarized in Fig. 1. All correlations were statistically significant (*p* < 0.001). In Sample 1, correlations ranged from 0.24 (Demonic–Ultimate Meaning) to 0.68 (Divine–Doubt). In Sample 2 (T1), correlations ranged from 0.13 (Demonic–Interpersonal) to 0.68 (Divine–Ultimate Meaning).

Across both samples, factors were moderately to highly intercorrelated. The strongest associations were observed between Divine and Doubt (φ = 0.68, 0.55), Divine and Ultimate Meaning (φ = 0.65, 0.68), and Moral and Doubt (φ = 0.66, 0.46). The weakest correlations involved the Demonic factor, particularly with Interpersonal struggles and Ultimate Meaning struggles.

### Internal Consistency

Internal consistency coefficients for all RSS-14 subscales and criterion measures are reported in Table S1. In Sample 1 (*N* = 593), McDonald’s ω values for the six RSS-14 subscales ranged from 0.67 (Interpersonal) to 0.83 (Divine), and Cronbach’s α values ranged from 0.57 (Interpersonal) to 0.77 (Divine). For the two-item subscales (Demonic, Moral, Doubt, Ultimate Meaning), Spearman–Brown split-half reliabilities ranged from 0.68 (Moral) to 0.84 (Ultimate Meaning).

In Sample 2 Time 1 (*N* = 355), McDonald’s ω values ranged from 0.70 (Interpersonal) to 0.78 (Divine), and Cronbach’s α ranged from 0.56 (Interpersonal) to 0.73 (Divine). Spearman–Brown reliabilities for two-item subscales ranged from 0.61 (Moral) to 0.81 (Demonic).

Across both samples, all subscales met or closely approached the 0.70 criterion typically recommended for research use. The Spearman–Brown reliabilities for two-item subscales exceeded or approximated established benchmarks for brief scales (Eisinga et al., 2013).

### Test–Retest Reliability

Test–retest reliability was examined in Sample 2 across three intervals over the six-week period (baseline = T1; 2.5–3 weeks = T2; 5–6 weeks = T3). Table S5 reports intraclass correlation coefficients ICC(A,1), standard errors of measurement (SEM), smallest detectable change at 95% confidence (SDC_95_), and Bland–Altman agreement statistics for each RSS-14 subscale.

Short-Term Stability (T1–T2). Over the initial 2.5–3 week interval, ICC(A,1) values ranged from 0.49 (Moral) to 0.63 (Demonic, Interpersonal), indicating moderate short-term stability. Mean differences between waves were minimal, ranging from − 0.23 (Moral) to − 0.04 (Demonic). Bland–Altman 95% limits of agreement varied across subscales (e.g., Divine: − 1.01 to 0.89; Moral: − 2.17 to 1.71), reflecting individual variability. A proportional bias effect was detected for Interpersonal struggles (*p* < 0.001); no other subscale showed significant bias.

Mid-Term Stability (T2–T3). Across the following ~ 2.5-week interval, ICCs improved slightly, ranging from 0.63 (Interpersonal) to 0.69 (Demonic). SEM values decreased (0.26–0.61), corresponding to smaller SDC₉₅ values (0.73–1.68). Mean differences were negligible (− 0.09 to + 0.14). Proportional bias tests were significant for Interpersonal (*p* = 0.020) and marginal for Demonic (*p* = 0.055); no other subscales showed significant bias.

Long-Term Stability (T1–T3). Across the full 6-week period, ICCs declined as expected with the longer interval, ranging from 0.44 (Moral) to 0.62 (Demonic, Ultimate Meaning), indicating low-to-moderate stability. SEM values ranged from 0.35 to 0.74 (SDC₉₅ = 0.98–2.06). Mean differences remained small (− 0.31 to + 0.07). Significant proportional bias was observed for Divine (*p* = 0.013) and Demonic (*p* = 0.018) subscales; no bias emerged for others.

### Measurement Invariance

Measurement invariance of the RSS-14 was evaluated across three waves of Sample 2 (T1: *N* = 355; T2: *N* = 307; T3: *N* = 251) using multi-group CFA with WLSMV estimation for ordinal indicators. Model-fit indices and comparisons are presented in Table 4.

**Table 4:** Measurement invariance across time points (Sample 2)

Model	χ^2^	*df*	CFI	RMSEA (90% CI)	SRMR	ΔCFI	ΔRMSEA	ΔSRMR	Δχ^2^	*Δdf*	*p*	Decision
Configural	390.25	186	0.974	0.062 [0.053, 0.071]	0.067	–	–	–	–	–	–	Baseline model
Metric	372.88	202	0.979	0.054 [0.046, 0.063]	0.070	+ 0.004	–0.008	+ 0.003	16.23	16	0.437	Supported
Scalar	451.97	260	0.976	0.051 [0.043, 0.059]	0.070	–0.003	–0.004	–0.000	79.13	58	0.034	Partially supported

Estimator = WLSMV; indicators treated as ordered; parameterization = delta; std.lv = TRUE. Invariance evaluated with scaled Δχ^2^ tests (Liu et al., 2017); nonsignificant Δχ^2^ (*p* ≥ 0.05) supports invariance. Wave Ns: T1 (*N* = 355), T2 (*N* = 307), T3 (*N* = 251). Final category structure after collapsing low-frequency categories is described in text. Strict invariance was not tested (full scalar invariance not established)

The configural model, establishing the six-factor structure at each time point, fit the data well: χ^2^(186) = 390.25, *p* < 0.001; CFI = 0.974; RMSEA = 0.062 [90% CI: 0.053, 0.071]; SRMR = 0.067. Imposing metric invariance (equal factor loadings across T1–T3) did not degrade fit: scaled Δχ^2^(16) = 16.23, *p* = 0.437; changes in practical fit indices were negligible (ΔCFI = + 0.004; ΔRMSEA = − 0.008).

Adding equality constraints on item thresholds (scalar invariance) yielded χ^2^(260) = 451.97, *p* < 0.001; CFI = 0.976; RMSEA = 0.051 [.043, 0.059]; SRMR = 0.070. Although the scaled Δχ^2^(58) = 79.13, *p* = 0.034, reached significance, practical fit changes remained within recommended thresholds (ΔCFI = − 0.003; ΔRMSEA = − 0.004; Chen, 2007). Scalar invariance was thus considered adequately supported in practical terms.

### Construct Validity

#### Convergent and Criterion Validity

Spearman correlations between RSS-14 subscales and external constructs are shown in Table 5. Across both samples, Negative Religious Coping correlated positively with all RSS-14 subscales (Sample 1: *ρ* = 0.26–0.64; Sample 2: *ρ* = 0.25–0.66; all *p* < 0.001), confirming that the RSS-14 captures constructs conceptually aligned with maladaptive religious coping responses. In contrast, Positive Religious Coping showed near-zero or weak associations (*ρ* ≤ 0.23), demonstrating appropriate discriminant validity from adaptive coping.

**Table 5:** Correlations between RSS-14 subscales and external validity measures

Scale/subscale	Divine	Demonic	Interpersonal	Moral	Doubt	Ultimate meaning
**Sample 1** **(*N*** **= 593)**						
*B-RCOPE (Religious Coping)*						
Positive Religious Coping	0.07	0.19***	0.04	0.12**	0.00	−0.03
Negative Religious Coping	0.64***	0.42***	0.26***	0.36***	0.44***	0.46***
*CRS-5* *(Centrality of Religiosity)*	−0.18***	0.10*	0.01	0.02	−0.18***	−0.24***
*S-DSES-7* (*Spiritual Experiences)*	−0.26***	−0.02	−0.07	−0.15***	−0.33***	−0.27***
*S-DSES-4* *(Divine Closeness)*	−0.21***	0.02	−0.03	−0.08*	−0.26***	−0.23***
*DASS-21 (Mental Health)*						
General Distress	0.53***	0.34***	0.21***	0.42***	0.32***	0.46***
Depression	0.53***	0.26***	0.18***	0.41***	0.36***	0.61***
Anxiety	0.40***	0.31***	0.18***	0.32***	0.23***	0.29***
Stress	0.47***	0.32***	0.21***	0.38***	0.28***	0.30***
*SWLS* *(Life Satisfaction)*	−0.28***	−0.14***	−0.11**	−0.25***	−0.17***	−0.38***
**Sample 2 (T1, *N*** **= 323)**						
*B-RCOPE (Religious Coping)*						
Positive Religious Coping	0.02	0.23***	−0.02	0.13*	−0.08	−0.08
Negative Religious Coping	0.66***	0.55***	0.25***	0.36***	0.28***	0.36***
*CRS-5* *(Centrality of Religiosity)*	−0.08	0.29***	0.04	0.09	−0.19***	−0.13*
*S-DSES-4* *(Divine Closeness)*	−0.19***	0.23***	−0.08	−0.03	−0.26***	−0.27***
*PHQ-4 (Mental Health)*						
Depression	0.38***	0.21**	0.42***	0.34***	0.39***	0.41***
Anxiety	0.39***	0.27**	0.39***	0.42***	0.34***	0.38***
*PSS-4* *(Perceived Stress)*	0.35***	0.13*	0.31***	0.30***	0.36***	0.34***
*WHO-5* *(Well-being)*	−0.29***	−0.07	−0.24***	−0.21***	−0.24***	−0.30***

Spearman’s *ρ*, two-tailed tests; pairwise complete observations. * *p* < 0.05, ** *p* < 0.01, *** *p* < 0.001. Stars indicate significance

Correlations with religiousness and positive spiritual experience were small or negative, as expected. In Sample 1, Centrality of Religiosity (CRS-5) correlated negatively with Divine (*ρ* = −0.18), Doubt (*ρ* = −0.18), and Ultimate Meaning (*ρ* = −0.24; all *p* < 0.001), and near zero with other subscales (*ρ* ≤ 0.10). Daily Spiritual Experiences (S-DSES-7) correlated negatively with Divine, Moral, Doubt, and Ultimate Meaning (*ρ* = −0.15 to −0.33, *p* < 0.001), though not significantly with Demonic or Interpersonal. In Sample 2, CRS-5 and SDSES-4 showed similar patterns, with one notable exception: both correlated positively with Demonic struggles (*ρ* = 0.23–0.29, *p* < 0.001).

#### Divergent and Discriminant Validity

RSS-14 subscales showed expected associations with mental health indicators, supporting criterion validity. In Sample 1, all subscales correlated positively with DASS-21 General Distress (*ρ* = 0.21–0.53, *p* < 0.001) and negatively with Life Satisfaction (*ρ* = −0.11 to −0.38, *p* ≤ 0.01). In Sample 2, subscales correlated positively with PHQ-4 Depression (*ρ* = 0.21–0.42), PHQ-4 Anxiety (*ρ* = 0.27–0.42), and Perceived Stress (*ρ* = 0.13–0.36; all *p* ≤ 0.05). Correlations with WHO-5 were negative for five subscales (*ρ* = −0.21 to −0.30, *p* < 0.001); the Demonic subscale showed a nonsignificant association (*ρ* = −0.07).

## Discussion

This study provides the first psychometric validation of the RSS-14 in Russian Orthodox Christian women. Across two independent samples of Russian Orthodox Christian women, the Russian adaptation of RSS-14 demonstrated acceptable-to-good psychometric properties. The hypothesized six-factor structure was confirmed, internal consistency was generally satisfactory despite subscale brevity, and measurement invariance analyses supported meaningful longitudinal comparisons. Construct validity was evidenced by theoretically consistent correlations with religious coping, psychological distress, religiosity, and well-being. These findings establish the RSS-14 as a valid and reliable instrument for assessing multidimensional R/S struggles among Russian Orthodox women.

### Descriptive Patterns

Moral struggles showed the highest mean scores, consistent with the ascetic tradition of Eastern Orthodoxy, which emphasizes continual repentance and self-examination (Mitralexis & Kaethler, 2023). Ultimate Meaning struggles were the second most endorsed domain, suggesting that existential concerns about life’s deeper purpose are salient in this population. Divine struggles and Doubt were less prominent, while Demonic struggles were moderate—contrasting with more secular Western samples where demonic struggles are often negligible (Lampe et al., 2021). Interpersonal struggles showed the lowest means; future qualitative research should explore whether Orthodox interpersonal religious conflicts manifest differently than the current items capture.

### Structural Validity

The six-factor model replicated across both samples, aligning with the original U.S. validation (Exline et al., 2023) and subsequent adaptations in Poland (Falewicz et al., 2023) and Portugal (Tomás & Moreira, 2024). Factor correlations indicated related but distinguishable constructs, mirroring patterns observed in prior studies (Smith et al., 2025). The absence of a well-fitting general factor likely reflects the RSS-14 development process, in which items were selected to maximize content breadth across six conceptually distinct domains rather than to maximize psychometric fit (Exline et al., 2023). The moderate interfactor correlations (φ = 0.13–0.68) indicate that while struggle domains share common variance, each captures unique experiential content supporting subscale-level interpretation.

Dynamic fit indices confirmed that observed fit was consistent with correct model specification (Table S4). All factor loadings exceeded the 0.40 criterion, though one item (Interpersonal 2) showed marginally lower loadings consistent with patterns in prior adaptations (Falewicz et al., 2023). The overall factor structure remained robust and theoretically coherent, suggesting the multidimensional struggles framework is applicable in Orthodox Christian contexts.

### Reliability and Measurement Precision

Internal consistency was acceptable-to-good given the scale’s brevity, aligning closely with prior RSS-14 validations (Exline et al., 2023; Tomás & Moreira, 2024). The Interpersonal subscale consistently shows lower reliability across studies, presumably due to item heterogeneity, yet remains acceptable for research and screening applications (Eisinga et al., 2013).

Test–retest reliability over the six-week period showed moderate stability with expected attenuation over longer intervals, suggesting RSS-14 scores reflect relatively state-like phenomena that fluctuate in response to life circumstances and religious contexts (Exline et al., 2023; Pargament & Exline, 2021). The substantial smallest detectable change values indicate that the RSS-14 is better suited for group-level research and clinical screening than individual-level change monitoring.

### Construct Validity

Convergent validity was robustly supported. Negative religious coping showed strong positive associations with all RSS-14 subscales, with Divine struggles showing the strongest relationship—replicating the original validation (Exline et al., 2023). Positive religious coping showed near-zero or weak associations, demonstrating appropriate discriminant validity. Demonic struggles correlated positively with both positive religious coping and religiosity, a pattern consistently observed in prior RSS validation studies (Exline et al., 2014, 2023) and likely reflecting that more religious individuals hold stronger beliefs in demonic forces.

Centrality of Religiosity correlated negatively with Divine, Doubt, and Ultimate Meaning struggles, indicating that greater religious salience is associated with fewer existential and theodicy-related struggles. However, minimal associations with Demonic, Interpersonal, and Moral struggles suggest these domains occur independently of overall religiosity level. This pattern diverges somewhat from Polish findings, where Interpersonal struggles showed clearer negative associations with religious centrality (Falewicz et al., 2023), highlighting potential cultural differences in how relationships between ecclesial conflicts and religious commitment manifest across Orthodox and Catholic contexts.

We hypothesize three potential explanations. First, Orthodox ecclesiology emphasizes mystical unity with the Church as the Body of Christ (Mitralexis & Kaethler, 2023), potentially allowing interpersonal conflicts with individuals to coexist with deep sacramental commitment. Second, Russian Orthodox culture may more strongly discourage criticism of ecclesiastical authority, suppressing overt interpersonal conflict while channeling tensions into moral self-criticism or internalized doubt. Third, Polish Catholicism’s distinct Church-state history may more directly link institutional dissatisfaction to personal religiosity. Cross-cultural studies using identical measures would help clarify these patterns.

All RSS-14 subscales correlated positively with psychological distress and negatively with life satisfaction and well-being. These magnitudes align with meta-analytic estimates (Smith et al., 2025) and longitudinal evidence that R/S struggles predict increases in depression and anxiety (Bockrath et al., 2022; Cowden et al., 2022). The stronger associations for Divine, Moral, and Ultimate Meaning struggles are consistent with theoretical models emphasizing these domains as particularly psychologically consequential (Exline et al., 2024).

### Measurement Invariance

Full metric invariance across three longitudinal waves was supported, enabling valid comparisons of relationships between RSS-14 subscales and external constructs over time (Putnick & Bornstein, 2016). Scalar invariance was supported based on practical fit criteria (ΔCFI < 0.010, ΔRMSEA < 0.015; Chen, 2007), though the scaled Δχ^2^ reached statistical significance—a common occurrence with ordered categorical data in moderate samples (Liu et al., 2017). Latent mean comparisons across waves are thus interpretable at the group level, though some threshold parameters may have shifted over the assessment period.

### Clinical and Pastoral Implications

The RSS-14 can serve as a brief screening tool to identify spiritually-based distress that might otherwise remain unaddressed. Given that R/S struggles predict unique variance in psychological distress beyond traditional risk factors (Cowden et al., 2022; Smith et al., 2025), mental health professionals working with Russian Orthodox clients should routinely assess for spiritual struggles. The multidimensional profile allows clinicians to tailor interventions to specific struggle domains (Pargament & Exline, 2022).

For pastoral caregivers, the consistent correlations between struggles and psychological distress underscore the need to recognize when parishioners require referral to mental health professionals. Conversely, mental health providers should consider pastoral consultation when R/S struggles are prominent. Collaborative care models integrating psychological and spiritual support need to be established in Russian Orthodox settings.

### Limitations and Future Directions

This study’s strengths include two independent samples with differing designs, rigorous psychometric methodology, comprehensive validity assessment, and application of dynamic fit indices. Several limitations warrant acknowledgment. First, both samples consisted exclusively of women; generalization to men should proceed with caution since R/S struggles manifest differently and relate to distress through distinct mechanisms across genders (e.g., Captari et al., 2022). Second, online convenience sampling may have introduced selection bias toward more educated and urban participants. Third, the Interpersonal subscale’s lower reliability suggests this domain may require item refinement. Fourth, moderate test–retest reliability indicates that the scale is better suited for group comparisons than precise individual monitoring. Finally, data collection during Great Lent may limit generalizability to non-Lenten periods; replication across diverse contexts is warranted.

## Conclusion

The Russian RSS-14 demonstrates acceptable-to-good psychometric properties for assessing multidimensional spiritual struggles in Russian Orthodox women. The six-factor structure replicated across samples, and validity evidence supported theoretically expected relationships with distress, religiosity, and coping. These findings fill a critical gap in Russian-language R/S assessment and provide a foundation for integrating spiritual and psychological dimensions in research and practice. The final Russian RSS-14 is available in Supplement S6.

## Supplementary Information

Below is the link to the electronic supplementary material.

Supplementary file1 (DOCX 35 kb)

## Data Availability

Data supporting the findings of this study are available from the corresponding author upon reasonable request.
