# Supplementary material for: Adaptation and Validation of the 14-Item Religious and Spiritual Struggles Scale Among Russian Orthodox Christian Women
Source: J Relig Health. 2026 Apr 9;65(4):3743–69. doi: 10.1007/s10943-026-02645-y (PMC13481397; doi:10.1007/s10943-026-02645-y)
Supplement: Supplementary file 1 [file 10943_2026_2645_MOESM1_ESM.docx]

# Supplementary Materials

**Table S1**
*Descriptive Statistics and Reliability Estimates for Study Measures*

| **Scale/Subscale (Range)** | ***M*** | ***SD*** | **ω** | **α** | ***ρSB*** |
| --- | --- | --- | --- | --- | --- |
| **Sample 1 (*N* = 593)** | | | | | |
| **RSS-14** (1–5) |  |  |  |  |  |
| Divine | 1.74 | 0.87 | .83 | .77 | — |
| Demonic | 1.77 | 0.91 | — | — | .78 |
| Interpersonal | 1.72 | 0.73 | .67 | .57 | — |
| Moral | 2.60 | 1.05 | — | — | .68 |
| Doubt | 1.92 | 0.97 | — | — | .77 |
| Ultimate Meaning | 2.17 | 1.15 | — | — | .84 |
| **B-RCOPE** (0–3) |  |  |  |  |  |
| Positive Religious Coping | 1.78 | 0.62 | .92 | .84 | — |
| Negative Religious Coping | 0.49 | 0.55 | .93 | .81 | — |
| **CRS-5** (1–5) | 4.26 | 0.51 | .84 | .64 | — |
| **SDSES-7** (1–6) | 3.85 | 0.92 | .93 | .88 | — |
| **DASS-21** (0–3) |  |  |  |  |  |
| General Distress | 1.24 | 0.51 | .96 | .93 | — |
| Depression | 1.14 | 0.60 | .92 | .86 | — |
| Anxiety | 0.96 | 0.57 | .91 | .85 | — |
| Stress | 1.61 | 0.58 | .95 | .89 | — |
| **SWLS** (1–7) | 3.71 | 1.18 | .86 | .81 | — |
| **Authentic Behavior** (1–5) | 2.70 | 0.59 | .88 | .79 | — |
| **Sample 2 (T1, *N* = 355)** | | | | | |
| **RSS-14** (1–5) |  |  |  |  |  |
| Divine | 1.34 | 0.55 | .78 | .73 | — |
| Demonic | 1.88 | 0.88 | — | — | .81 |
| Interpersonal | 1.61 | 0.70 | .70 | .56 | — |
| Moral | 2.77 | 0.98 | — | — | .61 |
| Doubt | 1.71 | 0.79 | — | — | .65 |
| Ultimate Meaning | 2.02 | 1.02 | — | — | .79 |
| **B-RCOPE** (0–3) |  |  |  |  |  |
| Positive Religious Coping | 1.81 | 0.59 | .91 | .83 | — |
| Negative Religious Coping | 0.43 | 0.47 | .90 | .75 | — |
| **CRS-5** (1–5) | 4.39 | 0.47 | .81 | .61 | — |
| **SDSES-4** (1–6) | 3.99 | 1.08 | .88 | .83 | — |
| **PHQ-4** (0–3) |  |  |  |  |  |
| Depression | 0.73 | 1.06 | — | — | .83 |
| Anxiety | 1.30 | 1.25 | — | — | .89 |
| **PSS-4** (0–4) | 1.77 | 0.77 | .87 | .78 | — |
| **WHO-5** (0–100) | 49.54 | 19.66 | .93 | .88 | — |

*Note.* ω = McDonald’s omega (primary); α = Cronbach’s alpha; *ρSB* = Spearman–Brown (for *k* = 2). Sample 2 (T1, *N* = 355). “—” indicates not applicable or not administered.

Table S2

*Data Screening, Exclusion, and Missingness Summary*

| **Characteristic** | **Sample 1 (*N* = 593)** | **Sample 2** | | |
| --- | --- | --- | --- | --- |
|  |  | **T1 (*N* = 355)** | **T2 (*N* = 307)** | **T3 (*N* = 251)** |
| **Missingness** | | | | |
| RSS-14 item-level missing (%) | 0.0 | < 0.8 | < 0.8 | < 0.8 |
| Wave-level attrition (%) | — | 25.2 | 34.9 | 47.3 |
| Maximum item-level refusal (%) | 2.2 | 5.6 | 4.5 | 3.6 |
| **Little's MCAR Test** | | | | |
| χ^²^ (*df*) | 0.12 (1) | — | 76.38 (53) | 13.20 (1) |
| *p* | .731 | — | .019 | < .001 |
| Conclusion | MCAR | — | MAR | MAR |

Note. Sample 1 = cross-sectional sample; Sample 2 = longitudinal sample. T1–T3 = assessments during Great Lent. Item-level missing = percentage of missing responses on individual RSS-14 items. Wave-level attrition = cumulative percentage of participants who did not complete the given wave. For Sample 1, Little's MCAR test was performed on composite-level validity measures. For Sample 2, the test was performed separately at each wave on available items. Em dash (—) indicates cross-sectional design (Sample 1) or insufficient testable missingness patterns (T1). MCAR = Missing Completely at Random; MAR = Missing at Random.

**Table S3**

*Item-Level Descriptive Statistics and Response Distributions*

| **Item** | ***M*** | ***SD*** | **Skew** | **Kurt** | **Missing %** | **Floor %** | **Ceiling %** |
| --- | --- | --- | --- | --- | --- | --- | --- |
| Di1 | 1.66 / 1.25 | 0.99 / 0.56 | 1.52 (.10) / 2.75 (.13) | 1.70 (.20) / 9.37 (.26) | .3% / .6% | 60.4% / 80.2% | 2.2% / .3% |
| Di2 | 1.82 / 1.42 | 1.11 / 0.78 | 1.20 (.10) / 2.15 (.13) | .49 (.20) / 4.86 (.26) | .3% / .6% | 56.0% / 70.8% | 3.2% / .8% |
| Di3 | 1.72 / 1.34 | 1.05 / 0.67 | 1.48 (.10) / 2.05 (.13) | 1.48 (.20) / 3.79 (.26) | .3% / .6% | 58.2% / 75.1% | 3.0% / 1.7% |
| De1 | 1.95 / 2.09 | 1.08 / 1.07 | 0.94 (.10) / 0.75 (.13) | 0.00 (.20) / -0.18 (.26) | .3% / 2.0% | 46.0% / 36.8% | 2.4% / 2.6% |
| De2 | 1.59 / 1.67 | 0.94 / 0.86 | 1.62 (.10) / 1.30 (.13) | 2.12 (.20) / 1.36 (.26) | .7% / 1.4% | 64.2% / 52.6% | 1.7% / .6% |
| IP1 | 1.51 / 1.38 | 0.96 / 0.88 | 1.98 (.10) / 2.58 (.13) | 3.13 (.20) / 6.36 (.26) | .2% / .3% | 72.3% / 78.8% | 1.9% / 2.3% |
| IP2 | 1.66 / 1.65 | 0.87 / 0.87 | 1.28 (.10) / 1.32 (.13) | 1.28 (.20) / 1.28 (.26) | .5% / .3% | 54.7% / 55.1% | .8% / .6% |
| IP3 | 1.98 / 1.78 | 1.15 / 1.08 | 1.04 (.10) / 1.38 (.13) | 0.12 (.20) / 1.11 (.26) | .3% / .3% | 45.9% / 55.4% | 4.1% / 3.4% |
| Mo1 | 2.61 / 2.80 | 1.12 / 1.11 | 0.29 (.10) / 0.06 (.13) | -0.68 (.20) / -0.67 (.26) | .7% / 2.0% | 17.7% / 14.1% | 5.4% / 6.3% |
| Mo2 | 2.59 / 2.76 | 1.29 / 1.20 | 0.38 (.10) / 0.27 (.13) | -0.99 (.20) / -0.75 (.26) | .5% / 1.4% | 23.9% / 16.6% | 9.5% / 10.6% |
| Do1 | 2.13 / 1.88 | 1.16 / 0.99 | 0.76 (.10) / 0.93 (.13) | -0.39 (.20) / 0.13 (.26) | .2% / .3% | 39.9% / 46.6% | 4.1% / 1.4% |
| Do2 | 1.71 / 1.53 | 0.99 / 0.83 | 1.39 (.10) / 1.61 (.13) | 1.32 (.20) / 2.02 (.26) | .3% / .8% | 56.5% / 64.2% | 1.9% / .3% |
| Me1 | 2.26 / 2.21 | 1.26 / 1.21 | 0.57 (.10) / 0.65 (.13) | -0.87 (.20) / -0.59 (.26) | .2% / 1.1% | 39.2% / 38.7% | 5.2% / 5.1% |
| Me2 | 2.08 / 1.83 | 1.22 / 1.03 | 0.86 (.10) / 1.08 (.13) | -0.87 (.20) / -0.46 (.26) | .3% / 1.2% | 36.7% / 23.9% | 9.3% / 9.8% |

***Note*.** Format: Sample 1 / Sample 2 (T1). Items presented as per their subscale assignments: Di = Divine Struggles; De = Demonic Struggles; IP = Interpersonal Struggles; Mo = Moral Struggles; Do = Doubt Struggles; Me = Ultimate Meaning Struggles M = mean; SD = standard deviation; Skew = skewness (SE); Kurt = kurtosis (SE). Floor/ceiling percentages represent endorsement of minimum (1) or maximum (5) response options on a 5-point scale. Sample 1 N = 593; Sample 2 (T1) N = 327.

**Table S4**
*Dynamic Fit Index (DFI) Cutoffs for RSS-14 Model*

|  | **CFI** | **RMSEA** | **SRMR** |
| --- | --- | --- | --- |
| Sample 1 DFI (L1, L2) | .97, .98 | .05, .04 | .17, .17 |
| Sample 2 (T1) DFI (L1, L2) | .95, .92 | .07, .09 | .19, .20 |
| Sample 2.2 DFI (L1, L2) | .98, .95 | .05, .07 | .14, .14 |
| Sample 2.3 DFI (L1, L2) | .98, .96 | .05, .07 | .13, .13 |

*Note.* DFI cutoffs derived from 500 Monte Carlo replications (McNeish & Wolf, 2023). L1 = Level 1 cutoff for minor misspecifications; L2 = Level 2 cutoff for major misspecifications. CFI = comparative fit index; RMSEA = root mean square error of approximation; SRMR = standardized root mean square residual.

**Table S5**

*Test–Retest Reliability (Sample 2 Longitudinal)*

| **Subscale** | ***N* (matched)** | **ICC(A,1) [95% CI]** | **SEM** | **SDC95** | **Mean diff** | **LoA (lower, upper)** | **Proportional bias (*p*)** |
| --- | --- | --- | --- | --- | --- | --- | --- |
| **T1–T2** | | | | | | | |
| Divine | 223 | 0.56 [0.47, 0.65] | 0.34 | 0.95 | −0.06 | −1.01, 0.89 | 0.900 |
| Demonic | 219 | 0.63 [0.55, 0.71] | 0.56 | 1.56 | −0.04 | −1.60, 1.52 | 0.230 |
| Interpersonal | 224 | 0.63 [0.54, 0.71] | 0.39 | 1.08 | −0.13 | −1.21, 0.94 | < .001 |
| Moral | 222 | 0.49 [0.38, 0.59] | 0.70 | 1.94 | −0.23 | −2.17, 1.71 | 0.851 |
| Doubt | 225 | 0.57 [0.48, 0.65] | 0.53 | 1.46 | −0.09 | −1.55, 1.37 | 0.543 |
| Ultimate Meaning | 224 | 0.60 [0.51, 0.68] | 0.66 | 1.82 | −0.08 | −1.90, 1.74 | 0.337 |
| **T2–T3** | | | | | | | |
| Divine | 182 | 0.66 [0.57, 0.73] | 0.26 | 0.73 | 0.01 | −0.73, 0.74 | 0.607 |
| Demonic | 180 | 0.69 [0.61, 0.76] | 0.54 | 1.49 | 0.14 | −1.35, 1.63 | 0.055 |
| Interpersonal | 183 | 0.63 [0.54, 0.71] | 0.37 | 1.01 | 0.00 | −1.01, 1.02 | 0.020 |
| Moral | 181 | 0.65 [0.55, 0.72] | 0.61 | 1.68 | −0.07 | −1.76, 1.61 | 0.574 |
| Doubt | 184 | 0.68 [0.59, 0.75] | 0.45 | 1.26 | 0.01 | −1.25, 1.27 | 0.345 |
| Ultimate Meaning | 183 | 0.66 [0.58, 0.74] | 0.60 | 1.67 | −0.09 | −1.76, 1.58 | 0.607 |
| **T1–T3** | | | | | | | |
| Divine | 199 | 0.46 [0.34, 0.56] | 0.35 | 0.98 | −0.08 | −1.06, 0.90 | 0.013 |
| Demonic | 194 | 0.62 [0.53, 0.70] | 0.60 | 1.67 | 0.07 | −1.60, 1.74 | 0.018 |
| Interpersonal | 200 | 0.60 [0.50, 0.68] | 0.41 | 1.14 | −0.12 | −1.26, 1.01 | 0.281 |
| Moral | 195 | 0.44 [0.31, 0.55] | 0.74 | 2.06 | −0.31 | −2.37, 1.75 | 0.515 |
| Doubt | 200 | 0.50 [0.38, 0.59] | 0.56 | 1.54 | −0.10 | −1.65, 1.44 | 0.552 |
| Ultimate Meaning | 200 | 0.62 [0.53, 0.70] | 0.61 | 1.68 | −0.09 | −1.77, 1.59 | 0.607 |

*Note.* ICC(A,1) = two-way mixed, absolute-agreement single-measure intraclass correlation; SEM = standard error of measurement; SDC95 = smallest detectable change (1.96√2 × SEM). LoA = Bland–Altman limits of agreement (95%). Mean difference = T2–T1 or T3–earlier. Proportional bias (slope test p-value) indicates whether mean and difference correlation is significant. ICC interpretation: < .50 = poor, .50–.75 = moderate, .75–.90 = good, > .90 = excellent.

**Supplement S6**
*The Russian Orthodox Religious and Spiritual Struggles Scale (RSS-14)*

**Шкала переживаний религиозного и духовного кризиса (RSS-14)**

**Инструкция для исследователей**

Возможно использование отдельных подшкал или адаптация инструкции под конкретное событие/временной период. При использовании шкалы в научных публикациях необходимо ссылаться на статью с валидацией текущей русской православной версии.

### Вступительная инструкция для респондентов

*«Многие люди в какие-то моменты жизни испытывают затруднения, беспокойство или сомнения, связанные с духовными или религиозными вопросами. В приведенном ниже списке утверждений нет правильных или неправильных ответов; лучший ответ – тот, который наиболее точно отражает Ваш опыт. Хотя в некоторых вопросах ниже используется слово "Бог", пожалуйста, при ответе подразумевайте то слово для обозначения Бога (или Высшей Силы), которое Вам ближе.*

*Пожалуйста, выбирайте вариант "совсем нет/не применимо" для тех утверждений, которые в рамках Вашего мировоззрения просто не имеют смысла.»*

### Общая инструкция

*«Пожалуйста, отметьте, в какой степени Вы испытывали нижеперечисленные переживания за последний месяц?»*

Также можно изменить временной промежуток: прошлый год, прошлая неделя, даже «*в то время*» или «*в настоящее время*» – тогда вы должны изменить формулировку с прошедшего на настоящее время.

### Ситуационная инструкция

Вы также можете использовать ситуационную инструкцию, как это делается в исследованиях совладаюшего поведения. Например, попросите людей вспомнить недавнее стрессовое событие (например, самое стрессовое событие за прошлый месяц, прошлый год или то, с которым они сталкиваются прямо сейчас). Затем задайте эти вопросы применительно к этому событию.

Например: *«В ответ на это конкретное событие, возникли ли у Вас какие-либо из следующих переживаний?»* Или, если все участники сталкиваются с общим стрессором: *«В ответ на известие о постановке диагноза, возникли ли у Вас нижеследующие переживания?»*

|  | **«*В течение последнего [месяца] я...*»** | **Совсем нет/не применимо**  **(1)** | **Немного**  **(2)** | **В некоторой степени**  **(3)** | **Довольно сильно**  **(4)** | **Сильно**  **(5)** |
| --- | --- | --- | --- | --- | --- | --- |
| **A** | Переживал(а) смятение и тревогу из-за сомнений в вере или духовной жизни | ☐ | ☐ | ☐ | ☐ | ☐ |
| **B** | Испытывал(а) чувство обиды или негодования по отношению к Богу | ☐ | ☐ | ☐ | ☐ | ☐ |
| **C** | Переживал(а), что служители Церкви или другие верующие плохо со мной обошлись, оскорбили или обидели меня | ☐ | ☐ | ☐ | ☐ | ☐ |
| **D** | Ощущал(а), что бесы или дьявол искушают или нападают на меня | ☐ | ☐ | ☐ | ☐ | ☐ |
| **E** | Задавался(-ась) вопросом, действительно ли моя жизнь имеет смысл | ☐ | ☐ | ☐ | ☐ | ☐ |
| **F** | Конфликтовал(а) или спорил(а) с другими людьми по вопросам веры и духовной жизни | ☐ | ☐ | ☐ | ☐ | ☐ |
| **G** | Боролся(-ась) с собой, в попытках следовать моим нравственным принципам | ☐ | ☐ | ☐ | ☐ | ☐ |
| **H** | Переживал(а) несогласие или раздражение по поводу устройства жизни Церкви или поступков её служителей | ☐ | ☐ | ☐ | ☐ | ☐ |
| **I** | Испытывал(а) неуверенность или колебался(-ась) в своих религиозных убеждениях | ☐ | ☐ | ☐ | ☐ | ☐ |
| **J** | Переживал(а), словно Бог меня наказывает | ☐ | ☐ | ☐ | ☐ | ☐ |
| **K** | Ощущала(а), что трудности, с которыми я сталкиваюсь, – это происки бесов или дьявола | ☐ | ☐ | ☐ | ☐ | ☐ |
| **L** | Переживал(а) ощущение, словно Господь оставил меня | ☐ | ☐ | ☐ | ☐ | ☐ |
| **M** | Переживал(а), что моя жизнь лишена особого смысла | ☐ | ☐ | ☐ | ☐ | ☐ |
| **N** | Испытывал(а) вину за то, что не удается жить согласно своим нравственным принципам | ☐ | ☐ | ☐ | ☐ | ☐ |

Подсчёт результатов

Результаты оцениваться путем вычисления среднего значения по пунктам подшкал (не путем суммирования).

| **Подшкала** | **Пункты** |
| --- | --- |
| Переживания кризиса в отношении Бога | B, J, L |
| Переживания кризиса, связанного со злыми силами | D, K |
| Переживания межличностного кризиса | C, F, H |
| Переживания нравственного кризиса | G, N |
| Переживания кризиса веры | A, I |
| Переживания кризиса смысла жизни | E, M |
